# Novel Role of Long Non-Coding RNA ASAP1-IT1 in Progression of Hepatocellular Carcinoma

**DOI:** 10.3389/fonc.2022.746896

**Published:** 2022-05-30

**Authors:** Yanping Liu, Chengguang Hu, Xiaoyong Qu, Honghui Chen, Logen Liu, Linlin Zhou, Side Liu, Guoqing Li, Yuanping Zhou

**Affiliations:** ^1^Department of Gastroenterology, Nanfang Hospital, Southern Medical University, Guangzhou, China; ^2^Department of Gastroenterology, Second Affiliated Hospital, University of South China, Hengyang, China; ^3^Department of Infectious Diseases and Hepatology Unit, Nanfang Hospital, Southern Medical University, Guangzhou, China; ^4^Department of Hepatobiliary and Pancreatic Surgery, Second Affiliated Hospital, University of South China, Hengyang, China; ^5^Clinical Research Center, The Second Affiliated Hospital, University of South China, Hengyang, China; ^6^Key Laboratory for Molecular Diagnosis and Precision Medicine in Hengyang, The Second Affiliated Hospital, University of South China, Henyang, China

**Keywords:** hepatocellular carcinoma, long non-coding RNA, cell proliferation, metastasis, prognosis, miR-221-3p

## Abstract

The long non-coding RNA (lncRNA) ASAP1-IT1 has been recently shown to aberrantly increase in ovarian and bladder cancer, while its role in other malignancies remains unexplored. This study was to characterize the expression and assess the potential role of ASAP1-IT1 in hepatocellular carcinoma (HCC). Fifty-four paired HCC and histologically normal tissues were obtained from HCC patients. Human HCC cell lines (HepG2, Huh7, SMMC-7721, and BEL-7402) and a normal liver cell line (LO2) were used for *in vitro* studies. ASAP1-IT1-specific siRNAs were used to silence ASAP1-IT1 expression, while the pcDNA-ASAP1-IT1 vector was constructed to up-regulate its expression. In situ hybridization and qRT-PCR were performed to characterize subcellular localization and expression of ASAP1-IT1. Cell proliferation and migration assays were conducted to examine the role of ASAP1-IT1 in the progression of HCC. In silico analysis was conducted to predict putative miRNA binding sites, which were validated by luciferase reporter assays. ASAP1-IT1 levels were significantly increased in HCC tissues and cells compared with controls. Notably, higher ASAP1-IT1 levels were significantly associated with poorer prognosis of HCC patients. In situ hybridization analysis revealed that ASAP1-IT1 was mainly localized in the nucleus of hepatoma cells and differentially expressed in trabecular, compact, and pseudoglandular forms of liver cancer. Furthermore, knockdown of ASAP1-IT1 significantly suppressed cell proliferation and migration, while its overexpression significantly promoted cell proliferation and migration of HCC cells. Mechanistically, ASAP1-IT1 might exert its role in HCC progression, at least in part, by directly interacting with miR-221-3p. In conclusion, ASAP1-IT1 is abnormally elevated in HCC, and higher levels are correlated with poorer prognosis. An underlying mechanism has been proposed for ASAP1-IT1-associated promotion of proliferation and migration in HCC cells. These findings have provided evidence supporting the oncogenic role of ASAP1-IT1 in HCC.

## Introduction

Liver cancer is among the most common malignancies in the world, with an incidence ranked sixth among all diagnosed cancers, and is the fourth most frequent cause of cancer-related deaths worldwide (1). Hepatocellular carcinoma (HCC) represents the predominant form of primary liver cancer, accounting for approximately 90% of all liver cancer patients (2). Hepatitis B virus (HBV) and hepatitis C virus (HCV) infections are the leading causes of HCC development. Although substantial progress and advances have been made regarding the diagnosis and the treatment of HCC (e.g. local ablation, surgical resection, liver transplantation, systemic therapies, and transarterial chemoembolization), the clinical outcome, such as five-year overall survival (OS), remains poor in patients with HCC worldwide (3–5). Until now, the molecular mechanisms underlying the pathogenesis and progression of HCC have been largely unknown. Therefore, the elucidation of hepatocarcinogenesis mechanisms is required to guide the development of novel approaches to improve the clinical management of patients with HCC.

Long non-coding RNAs (lncRNAs) are an emerging class of non-coding RNAs (ncRNAs) that are longer than 200 bp. The recent decades have witnessed extensive studies in charactering the expression and understating of the function of lncRNAs. It has been shown that lncRNAs play critical roles in the regulation of gene expression at the transcriptional and post-transcriptional levels, through which lncRNAs are involved in multiple cellular processes, such as cell proliferation and migration. LncRNAs function as miRNA sponges or decoys to sequester miRNAs, thereby preventing their binding to target mRNAs (6). Extensive research has demonstrated that lncRNAs exert important roles in the carcinogenesis and development of cancer by either promoting or suppressing cancer growth and progression (7, 8). Therefore, lncRNAs that are aberrantly expressed in cancer have potential as markers for the prediction of susceptibility and prognosis, and as therapeutic targets in the development of novel cancer treatments.

Recently, the lncRNA ASAP1-IT1 (the intronic transcript 1 [IT-1] of ArfGAP with SH3 domain, ankyrin repeat and PH domain 1 [ASAP1]) was first identified as a novel lncRNA that was aberrantly increased in ovarian cancer. ASAP1-IT1 on chromosome 8q24.21 is 1179 bp in length. Fu and colleagues (9) have shown that the expression levels of ASAP1-IT1 are significantly correlated with the clinical outcomes, such as OS, of ovarian cancer patients. An additional study by Yang et al. (10) reported that ASAP1-IT1 is highly expressed in human bladder cancer tissues, and overexpression of ASAP1-IT1 in bladder cancer cells promotes the development of stem cell-like functions. In a recent study, the high expression of ASAP1-IT1 has been linked to the poor prognosis in cholangiocarcinoma (11). Until now, the potential role of ASAP1-IT1 in other malignancies, including HCC, remains unexplored.

In this study, the expression of ASAP1-IT1 was characterized, and its potential role in HCC was assessed using human HCC tissues and cell lines. The results obtained from this study will advance our understanding of lncRNA ASAP1-IT1 in HCC and may have clinical implications for the development of novel therapies and treatment options for HCC patients.

## Materials and Methods

### Human Tissue Samples

Fifty-four paired human HCC and adjacent, histologically normal tissues were collected from HCC patients who underwent surgical treatment during the period between January 2018 and December 2020 at the Second Affiliated Hospital, University of South China and Nanfang Hospital, Southern Medical University. The diagnosis of HCC was pathologically confirmed by experienced pathologists in the Department of Pathology at either hospital.

Prior to the study, all patients provided written informed consent. The study was approved by the Ethics Committee of the Second Affiliated Hospital, University of South China and Nanfang Hospital, Southern Medical University.

### Cell Lines and Culture

Four human HCC cell lines (HepG2, Huh7, SMMC-7721, and BEL-7402) and one normal liver cell line (LO2) as well as HEK293T cells were maintained in Dulbecco’s modified Eagle’s medium (DMEM) (Gibco, USA) supplemented with 10% fetal bovine serum (FBS; Gibco, USA) and 1% streptomycin-penicillin (100 U/mL penicillin and 100 μg/mL streptomycin; Invitrogen) in a humidified incubator maintained at 37°C with 5% CO2.

### Silencing of LncRNA ASAP1-IT1 in HCC Cell Lines

Silencing of lncRNA ASAP1-IT1 was performed using ASAP1-IT1-specific small interfering RNA (siRNA) in HCC cell lines. ASAP1-IT1-specific siRNA and non-specific control siRNA (NC-siRNA) were synthesized by Invitrogen (Carlsbad, CA, USA), and their sequences were as follows: ASAP1-IT1-specific siRNA1: 5’-GCUGCGACAAUAGACAUCGGAGUUU-3’; ASAP1-IT1-specific siRNA 2: 5’-CAGCACCCGAUGUCAUUCCUGGGAA-3’; ASAP1-IT1-specific siRNA3: 5’-UGAAGGCAGAGUGGUAGGCUCGGAA-3’; NC siRNA: 5’-UUCUCCGAACGUGUCACGUTT-3’. In brief, HCC cells were initially seeded in a 24-well plate. After reaching 70-80% confluency, the cells were transfected with ASAP1-IT1-specific siRNA or NC-siRNA using the transfection reagent Lipofectamine^®^ 2000 (Invitrogen, USA) according to the manufacturer’s instructions. At 48 h after transfection, the cells were harvested for subsequent analysis.

### Overexpression of LncRNA ASAP1-IT1 in HCC Cell Lines

For overexpression of lncRNA ASAP1-IT1 in HCC cells, the full-length human ASAP1-IT1 cDNA was amplified by PCR and inserted into the lentiviral vector. HCC cell lines were infected with different lentiviral vectors that stably expressed wild-type ASAP1-IT1, mutant ASAP1-IT1 (harboring mutant miR-221-3p-binding sites), or the empty vector as the scramble control using Lipofectamine^®^3000 (Invitrogen, Waltham, MA, USA) following the manufacturer’s protocol. Overexpression of ASAP1-IT1 was confirmed at 48 h post-transfection.

### RNA Extraction and qRT-PCR

Total RNA was extracted from human tissues or cells using the TRIzol reagent (Invitrogen, CA, USA) according to the manufacturer’s instructions, and was quantified using a Nanodrop 2000 (Wilmington, DE, USA). Total RNA (1 μg) was used as the template for cDNA synthesis using the cDNA Reverse Transcription kit (Applied Biosystems, Foster, CA, USA). The quantitative reverse transcription-polymerase chain reaction (qRT-PCR) assay was conducted using the Bio-Rad CFX96 Real-time PCR System (Bio-Rad, Foster, CA, USA). The reactions were incubated in a 96-well plate at 95°C for 5 min, followed by 39 cycles of 95°C for 10 s and 60°C for 34 s. The primer sequences used for qRT-PCR were as follows: ASAP1-IT1 (F) 5’- AAACATCATCCCCAGAGTGG-3’, and ASAP1-IT1 (R): 5’- GCCTTGCTCACCTCTGAAAC -3’. The expression levels of interest genes were calculated using the 2^−ΔΔCt^ method and normalized to the internal control gene glyceraldehyde 3-phosphate dehydrogenase (GAPDH).

### Cell Proliferation Assay

An MTT assay was performed to examine cell viability. In brief, cells were seeded in a 96-well plate (5000 cells per well). The MTT solution (5 mg/ml, 20 μl) was added to each well and the plate was incubated for 4 h. The cell viability was measured at 560 nm on a microplate absorbance reader.

For the colony formation assay, cells plated in a six-well plate (3000 cells per well) were cultured in DMEM supplemented with 10% FBS at 37°C for two weeks. Cells were then washed with phosphate-buffered saline (PBS), fixed using methanol for 0.5 h, stained with 1% crystal violet, and finally the colonies were counted.

### Cell Migration Assay

A Transwell™ assay was conducted to examine the ability of the cells to migrate. Briefly, cells were placed in the upper chamber of an insert (5 × 10^4^ cells per insert; 8 μm pore size; Millipore), and cell culture medium supplemented with 10% FBS was added to the lower chamber. After incubation for 24 h, cells remaining on the top of the membrane were removed using a cotton swab, while the cells that migrated onto the bottom of the membrane were fixed and stained with methanol and 0.1% crystal violet. The migrated cells were visualized and counted using an IX71 inverted microscope (Olympus, Tokyo, Japan).

### Wound Healing Assays

In the wound healing assays, hepatoma cells were grown as confluent monolayer cells in 6-well plates and were wounded by scraping with a 200-μl plastic pipette tip. The cells were then rinsed several times using culture medium to eliminate the wound-derived dislodged cells. The wound healing process was monitored and analyzed with an inverted light microscope (Olympus, Tokyo, Japan).

### *In Situ* Hybridization for ASAP1-IT1

Hepatoma cells ASAP1-IT1 localization and expression in human HCC tissues were analyzed using in situ hybridization with the RNA scope 2.5 HD-Brown Manual Assay (Advanced Cell Diagnostics, Newark, CA) according to the manufacturer’s instructions. The probes used in this study, including ASAP1-IT1, hs-PPIB (positive control, 313901), and dapB (negative control, 310043) were purchased from Advanced Cell Diagnostics. Expression levels of all probes were determined by staining with 3,3′-diaminobenzidine (DAB).

### Dual-Luciferase Reporter Assay

Putative binding sites for miR-221-3p in the 3’-UTR of ASAP1-IT1 were predicted by conducting an in silico analysis using the StarBase database. Wild-type and mutant DNA fragments were sub-cloned into the luciferase reporter vectors SV40-firefly_Luciferase-MCS (GV272, Genechem, China). The luciferase vectors and miR-221-3p mimics were co-transfected into HEK293T cells (1 × 10^4^ cells for each group) using Lipofectamine 3000. The Dual Luciferase Reporter Assay System (Promega, Madison, WI, USA) was used to analyze luciferase activity, which was recorded as the ratio of firefly luciferase activity to renilla luciferase activity. Experiments were performed in triplicate and each was repeated three times.

### Statistical Analysis

Statistical analysis was performed using Prism8.0 software (GraphPad, USA). Data were expressed as mean ± standard deviation (SD) of at least three independent assays. Student’s t-test and one-way analysis of variance (ANOVA) were used for comparative analyses of differences between groups. The Kaplan-Meier method (log-rank test) was used to generate survival curves for survival analysis. A p value less than 0.05 was considered statistically significant.

## Results

### ASAP1-IT1 Was Up-Regulated in Human HCC Tissues and Cell Lines, and Higher Levels Were Associated With a Poorer Prognosis of HCC Patients

**Figure 1:**
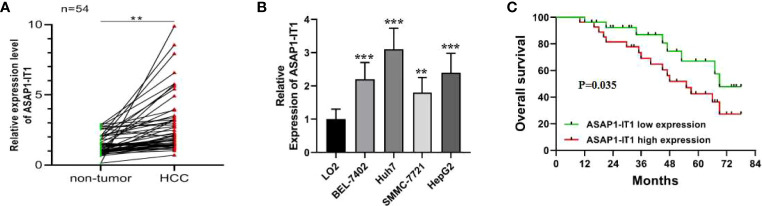
Dysregulation of ASAP1-IT1 in hepatocellular carcinoma (HCC) and its correlation with prognostic factors in HCC patients. **(A)** ASAP1-IT1 expression levels in HCC and matched adjacent normal liver tissues. Quantitative reverse transcription polymerase chain reaction (qRT-PCR) analysis revealed that ASAP1-IT1 expression was significantly elevated in HCC tissues versus control specimens (**p < 0.01). **(B)** ASAP1-IT1 expression levels in HCC cells and normal liver cells. qRT-PCR analysis revealed that ASAP1-IT1 expression levels were significantly higher in the four HCC cell lines, including HepG2, Huh7, SMMC-7721, and BEL-7402, compared with the normal liver cell line, LO2 (**p < 0.01, ***p < 0.001 vs LO2). **(C)** Kaplan-Meier survival analysis of correlation between ASAP1-IT1 expression and overall survival (OS) in HCC patients. Data were expressed as mean ± standard deviation (SD).

To understand the potential role of ASAP1-IT1 in HCC, the expression levels of ASAP1-IT1 were examined in 54 human HCC tissues and compared with matched histologically normal liver tissues, and four HCC cell lines were compared with a normal liver cell line. As shown in **Figure 1A**, the expression levels of ASAP1-IT1 were significantly elevated in HCC compared to control tissues. Similarly, the expression levels of ASAP1-IT1 were significantly higher in the four HCC cell lines compared to the normal liver cell line (**Figure 1B**). Notably, the highest levels of ASAP1-IT1 were in HepG2 and Huh7 cells (**Figure 1B**). Therefore, these two cell lines were used for subsequent studies to evaluate the function of ASAP1-IT1.

To determine the relationship between ASAP1-IT1 expression and clinical prognostic features of HCC patients, patients were followed-up for a median of 45.6 ± 20.1 months. The median expression level of ASAP1-IT1 was determined and used as the threshold for the classification of human HCC tissues into two groups: high ASAP1-IT1 expression group (n=27, 50%) and low ASAP1-IT1 expression group (n=27, 50%). As summarized in **Table 1**, higher levels of ASAP1-IT1 expression were significantly associated with higher Barcelona Clinic Liver Cancer (BCLC) stages (p = 0.028), a greater number of satellite nodules (p = 0.027), and poorer pathological differentiation (p = 0.013). However, there were no significant differences in age, gender, cause of liver disease, Child-Pugh score, tumor number, or vascular invasion (**Table 1**). The Kaplan-Meier survival analysis revealed that HCC patients in the high ASAP1-IT1 expression group had significantly lower OS rates than those in the low ASAP1-IT1 expression group (**Figure 1C**).

**Table 1:** The association between ASAP1-IT1 expression and clinicopathological features of HCC patients.

Variable	ASAP1-IT1 Expression		*p* value
	High (n = 27)	Low (n = 27)	
Age			0.782
< 60	15	17	
≥60	12	10	
Gender			1.000
Male	21	20	
Female	6	7	
Origin of liver disease			1.000
HBV	22	21	
Others	5	6	
Child-Pugh score			0.586
A	13	16	
B	14	11	
BCLC stage			0.028*
0/A	10	19	
B/C	17	8	
Tumor number			0.569
Solitary	16	19	
Multiple	11	8	
Satellite nodule			0.027*
Absent	11	20	
Present	16	7	
Vascular invasion			0.170
Absent	12	18	
Present	15	9	
Differentiation			0.013*
Poor	17	7	
Well/Moderate	10	20	

HBV, Hepatitis B Virus; BCLC, Barcelona Clinic Liver Cancer; HCC, hepatocellular carcinoma.

The data were analyzed by χ^2^ test or Fisher’s exact test, *p< 0.05.

### *In Situ* Hybridization for Characterization of ASAP1-IT1 Expression in HCC

**Figure 2:**
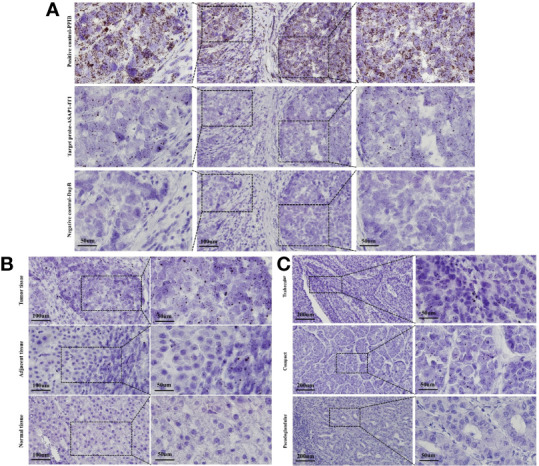
RNAscope in situ hybridization for characterization of subcellular localization and expression of ASAP1-IT1 in hepatocellular carcinoma (HCC) tissues. **(A)** Subcellular localization of ASAP1-IT1 in the nucleus of hepatoma cells. The specific probe for host gene PPIB was used as positive control, and non-specific probe DapB was used as the negative control. **(B)** Abnormal high expression of ASAP1-IT1 in human HCC tissues. **(C)** ASAP1-IT1 expression levels in different liver cancer types. ASAP1-IT1 was highly expressed in trabecular type (n=27, 50.0%), lowly expressed in compact type (n=21, 38.9%), and nearly undetectable in pseudoglandular type of HCC (n=6, 11.1%).

With RNA scope technology, a cutting-edge RNA in situ detection platform, in combination with specific probes for ASAP1-IT1, the subcellular localization and the expression of ASAP1-IT1 was characterized in human HCC and matched noncancerous tissues. As illustrated in **Figure 2A**, ASAP1-IT1 was mainly expressed in the nucleus of hepatoma cells. Furthermore, it was found that ASAP1-IT1 was abnormally elevated in human HCC tissues compared with the matched adjacent tissues, with nearly undetectable expression in normal liver tissues (**Figure 2B**). An analysis of the relationship between ASAP1-IT1 expression levels and types of liver cancer (e.g. trabecular, compact, pseudoglandular) noted a high expression in trabecular type (n=27, 50.0%), low expression in compact type (n=21, 38.9%), and nearly undetectable expression in pseudoglandular type HCC (n=6, 11.1%) (**Figure 2C**).

### Effects of ASAP1-IT1 on Proliferation of HepG2 and Huh7 Cells

**Figure 3:**
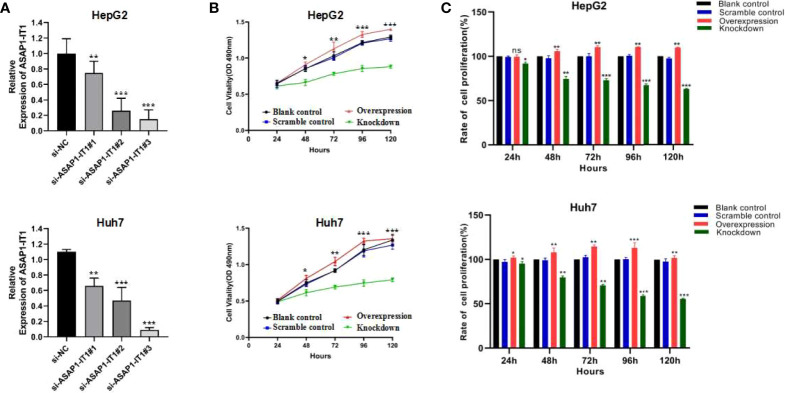
Effects of ASAP1-IT1 on cell proliferation in HepG2 and Huh7 cells. **(A)** Knock-down of ASAP1-IT1 in HepG2 and Huh7 cells transfected with ASAP1-IT1-specific small interfering (si)RNAs or nonspecific (NC) siRNA (si-NC) as a control in HepG2 (upper panel) and Huh7 (lower panel) cells. **(B)** MTT assay for cell viability. ASAP1-IT1 siRNA significantly suppressed cell viability compared with control groups, while overexpression of ASAP1-IT1 significantly increased cell viability in HepG2 (upper panel) and Huh7 (lower panel) cells. **(C)** Comparison of cell proliferation rates between groups in HepG2 (upper panel) and Huh7 (lower panel) cells. Data were expressed as mean ± standard deviation (SD) of at least three independent experiments. n.s., Not statistically significant; *p < 0.05, **p < 0.01, ***p < 0.001 vs control groups.

Intrigued by the interesting findings of ASAP1-IT1 in human HCC tissues and its prognostic value in HCC, the biological function of ASAP1-IT in HCC was further investigated via both silencing and overexpression of ASAP1-IT1 in cell lines. As shown in **Figure 3**, ASAP1-IT1-specific siRNAs were used to knockdown ASAP1-IT1 expression in HCC cells (**Figure 3A**), and si-ASAP1-IT1#3 elicited the greatest level of gene knockdown among the tested ASAP1-1T1 siRNAs in both HCC lines. Therefore, si-ASAP1-IT1#3was selected for subsequent functional studies. Overexpression of ASAP1-IT1 was achieved by transfection with the expression vector pcDNA-ASAP1-IT1. The MTT assay revealed that knockdown of ASAP1-IT1 resulted in a significant decrease in cell viability compared with the non-specific control, while overexpression of ASAP1-IT1 led to a significant increase of cell viability in HepG2 and Huh7 cells (**Figure 3B**). Moreover, cell proliferation rates were compared between groups (**Figure 3C**), and the results suggested that ASAP1-IT1-specific siRNAs significantly reduced proliferation while its overexpression promoted proliferation in HepG2 and Huh7 cells. These findings indicated a direct role for ASAP1-IT1 in HCC cell proliferation.

### Effects of ASAP1-IT1 on Cell Migration of HepG2 and Huh7 Cells

**Figure 4:**
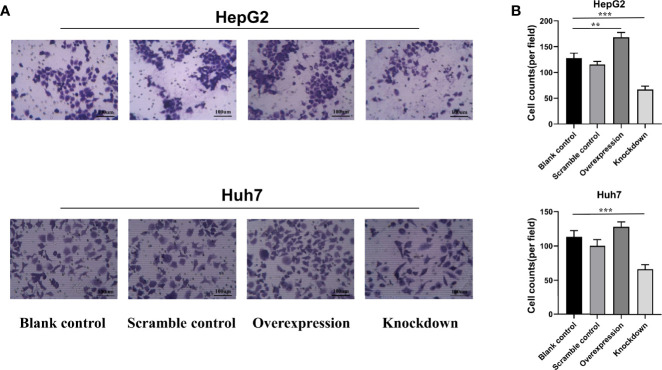
Transwell™ assay revealed effects of ASAP1-IT1 on cell migration in HepG2 and Huh7 cells. **(A)** Overexpression of ASAP1-IT1 promoted cell migration and down-regulation of ASAP1-IT1 inhibited cell migration in Huh7 and HepG2 cells compared with control groups. **(B)** Number of migrated cells per field. Data were expressed as mean ± standard deviation (SD) of at least three independent experiments. **p < 0.01, ***p < 0.001 vs control groups.

**Figure 5:**
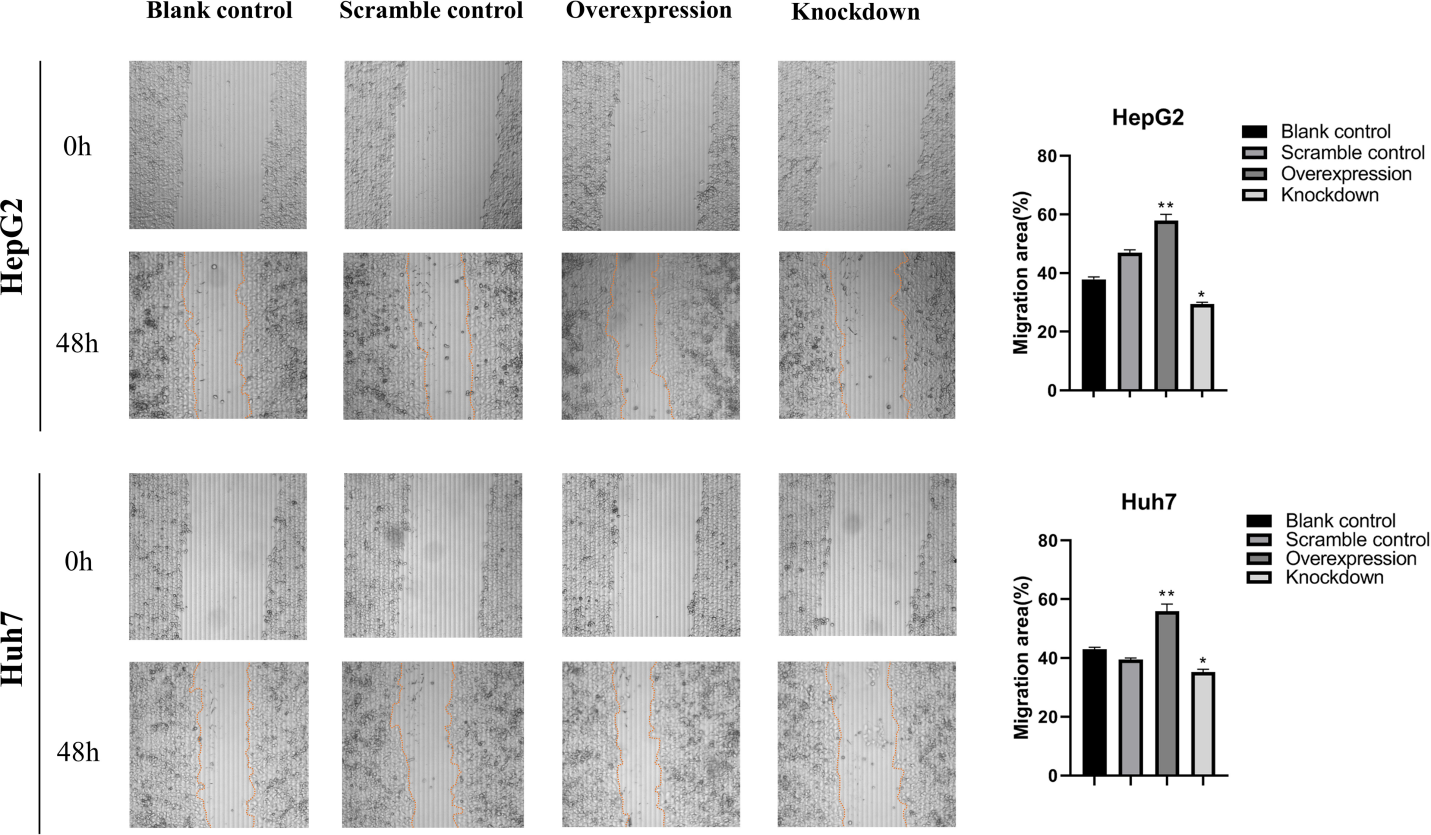
Scratch wound healing assays revealed effects of ASAP1-IT1 on cell migration in HepG2 and Huh7 cells. Scratch wound healing assays showed that knockdown of ASAP1-IT1 reduced the migration ability, while overexpression of ASAP1-IT1 promoted migration in HepG2 and Huh7 cells. The wound edge at 0 h, Scale bar, 200 μm. The distance migrated by the cells at the leading edge was quantitated and plotted at 48 h. The data were expressed as the means ± standard deviation (SD) from three independent experiments. *p < 0.05, **p < 0.01 vs control groups.

To further investigate the biological role of ASAP1-IT1, cell proliferation, Transwell, and scratch wound healing assays were performed in HepG2 and Huh7 cells with knockdown or overexpression of ASAP1-IT1. The migratory ability of HepG2 and Huh7 cells was suppressed in cells transfected with ASAP1-IT1-specific siRNAs compared with NC-siRNA (**Figure 4**). In contrast, HepG2 and Huh7 cells with overexpression of ASAP1-IT1 exhibited enhanced migratory capability (**Figure 4**). In parallel, the scratch wound healing assay revealed that knockdown of ASAP1-IT1 reduced migration, while overexpression of ASAP1-IT1 promoted migration of HepG2 and Huh7 cells (**Figure 5**). Collectively, these assays in HepG2 and Huh7 cells with knockdown or overexpression of ASAP1-IT1 confirmed direct effects of ASAP1-IT1 on cell migration.

### ASAP1-IT1 Acted as a Sponge of MiR-221-3p

**Figure 6:**
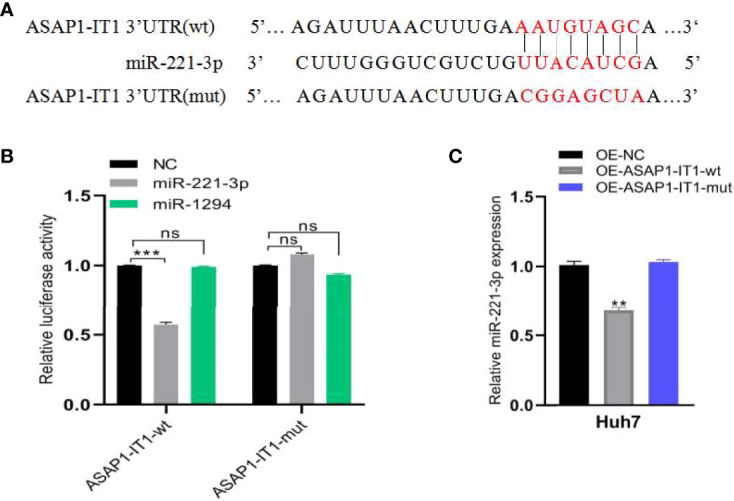
ASAP1-IT1 functions as a sponge for miR-221-3P. **(A)** Schematic representation of wild-type (wt) and mutant (mut) ASAP1-IT1 with mutations to the binding sites for miR-221-3p. **(B)** Luciferase activity of wt or mut ASAP1-IT1 in HEK293T cells after co-transfection with miR-221-3p mimics or NC mimics. **(C)** qRT-PCR analysis of relative expression levels of miR-221-3p in OE-NC, OE-ASAP1-IT1-wt, or OE-ASAP1-IT1-mut transfected Huh7 cells. All experiments were performed in triplicate and each was repeated three times. n.s. Not statistically significant; **p < 0.01, ***p < 0.001.

Next, the molecular mechanism of ASAP1-IT1 in HCC progression was investigated. In silico analysis predicted certain putative target miRNAs of ASAP1-IT1, including miR-221-3p and miR-1294, using StarBase and TargetScan databases. The putative binding sites between the ASAP1-IT13’-UTR and miR-221-3p (rather than miR-1294) were then validated by dual-luciferase reporter assays, in which miR-221-3p or miR-1294 mimics were co-transfected with ASAP1-IT1 luciferase reporters into HEK293T cells (**Figures 6A, B**). As shown in **Figure 6B**, the luciferase activity was significantly reduced by approximately 55% after overexpression of miR-221-3p but was not altered after miR-1294 overexpression. Notably, ectopic miR-221-3p expression significantly reduced the luciferase activity of the wild-type ASAP1-IT1 reporter (*p* < 0.05) but not the mutant reporter with mutant binding sites for miR-221-3p in 293T cells (**Figures 6A, B**). These findings provided several lines of evidence that ASAP1-IT1 can bind directly to miR-221-3p.

Furthermore, HCC cell models were constructed by transfecting OE-NC, OE-ASAP1-IT1-wt, and OE-ASAP1-IT1-mut in Huh7 cells. qRT-PCR analysis showed that the expression of miR-221-3p was down-regulated in OE-ASAP1-IT1-wt Huh7 cells, but not in OE-ASAP1-IT1-mut Huh7 cells (**Figure 6C**).

## Discussion

The major novel findings of this study are summarized as follows: 1) LncRNA ASAP1-IT1 was significantly increased in HCC tissues and cell lines compared with normal controls; 2) Higher ASAP1-IT1 expression was significantly correlated with poorer prognosis of HCC patients; 3) In situ hybridization analysis revealed the subcellular localization of ASAP1-IT1 to be the nucleus of hepatoma cells; 4) ASAP1-IT1 was differentially expressed in liver cancer types [highly expressed in trabecular type, lowly expressed in compact type, and nearly undetectable in pseudoglandular type]; 5) Knockdown of ASAP1-IT1 using specific siRNAs significantly suppressed cell proliferation and migration, whereas overexpression of ASAP1-IT1 significantly promoted cell proliferation and migration in HepG2 and Huh7 cells; and 6) *In silico* analysis and luciferase reporter assays revealed that ASAP1-IT1 acted as a sponge of miR-221-3P in HCC cells.

In the past decades, a great number of lncRNAs have been found to be dysregulated in malignant tumors, and multiple lines of scientific evidence have supported the regulatory role of lncRNAs in the development and progression of various cancers (12–14). It has been noted in previous studies that lncRNAs have the potential to be used as diagnostic and prognostic markers as well as therapeutic targets in a variety of tumors (15–20). With advanced human genomic technology, a growing number of lncRNAs have been identified in association with human cancers, including HCC. The lncRNA ASAP1-IT1 (*Homo sapiens* ASAP1 intronic transcript 1) was first identified as an aberrantly elevated lncRNA in ovarian cancer (9), and it can promote cell proliferation and metastasis of non-small cell lung cancer *via* modulation of the PTEN/AKT signaling axis (21). A previous study has indicated that the abnormally high expression of lncRNA ASAP1-IT1 can predict poor prognosis in patients with bladder cancer (10). In epithelial ovarian cancer, lncRNA ASAP1-IT1 has been shown to function as a modulator of gene expression (9). The high expression level of ASAP1-IT1 was also tested in Cholangiocarcinoma tissues and cells, ASAP1-IT1 could improve Cholangiocarcinoma progression and development *via* hedgehog signaling pathway (11). These previous studies have demonstrated that ASAP1-IT1 has pro-oncogenic properties. In this study, the prognostic value of lncRNA ASAP1-IT1 for HCC was first assessed, and the results suggested that high levels of ASAP1-IT1 were negatively correlated with the OS of HCC patients. Considering that nearly 80% (43/54) of HCC cases were attributable to HBV, it is worth further investigating whether the increased ASAP1-IT1 is specific to HBV-related HCC.

In the present study, RNA scope technology was used with specific probes to detect the expression of ASAP1-IT1 in HCC tissues, and the data revealed that it was mainly expressed in the nucleus of human hepatoma cells. Furthermore, ASAP1-IT1 has a high level of expression in HCC tissues, low level of expression in matched adjacent noncancerous tissues, and was generally not expressed in normal liver tissues. These results were consistent with qRT-PCR analysis results. With the RNA scope technology, the expression of ASAP1-IT1 was further examined in different types of liver cancer, and ASAP1-IT1 was found to be highly expressed in the trabecular type of liver cancer (n=27, 50.0%), lowly expressed in the compact type of liver cancer (n=21, 38.9%), and lowly or not expressed in the pseudoglandular type of liver cancer (n=6, 11.1%). Intrigued by the differential expression of ASAP1-IT1 in different types of liver cancer, in-depth investigations have been planned to clarify the cause of this difference and its potential clinical significance in a future study.

It merits attention that silencing of ASAP1-IT1 using specific siRNAs inhibited cell proliferation and migration, while overexpression of ASAP1-IT1 promoted these cellular processes in two HCC cell lines, HepG2 and Huh7. These findings indicated a biological significance of ASAP1-IT1 in promoting tumor cell proliferation and migration *in vitro*. These results also suggested a role for ASAP1-IT1 in the progression of HCC.

LncRNAs have emerged as regulators of gene expression through competing endogenous RNA networks (6). In this study, *in silico* analysis and luciferase assays were performed to explore the underlying mechanism of ASAP1-IT1 in HCC. The findings suggested that ASAP1-IT1 could bind directly to miR-221-3p. A number of previous studies reported that miR-221-3p was up-regulated in 70-80% of HCC cases, and exerted its role on HCC cell viability and motility *via* targeting the leukemia inhibitory factor receptor (LIFR) (22–24). Compared to OE-ASAP1-IT1-mut, OE-ASAP1-IT1-wt led to a decrease in miR-221-3p expression in the present study. Based on our findings and those of others, we propose that ASAP1-IT1 promotes HCC progression through sponging miR-221-3p. However, the study of ASAP1-IT1 in HCC is still in its infancy, and the biological functions of the ASAP1-IT1/miR-221-3p/LIFR axis in HCC progression require further in-depth investigation in future studies.

In conclusion, ASAP1-IT1 was abnormally increased in HCC and higher levels were correlated with tumor progression and metastasis. Notably, ASAP1-IT1 acted as a sponge of miR-221-3p in HCC cells. These novel findings have also provided evidence in support of the carcinogenic role for ASAP1-IT1 in HCC. Therefore, ASAP1-IT1 may serve as a molecular marker of poor prognosis to predict the OS of HCC patients, and as a therapeutic target for the development of new drugs or regimens to prevent the progression of HCC.

## Data Availability Statement

The raw data supporting the conclusions of this article will be made available by the authors, without undue reservation.

## Ethics Statement

The study was approved by the Ethics Committee of the Second Affiliated Hospital, University of South China and Nanfang Hospital, Southern Medical University. The ethics committee waived the requirement of written informed consent for participation.

## Author Contributions

GL and YZ designed the study. XQ, CH, LL, and LZ collected clinical samples and data, YL and CH carried out relevant experiments and analyzed data. YL, CH, SL, GL and YZ wrote and revised the manuscript. All authors contributed to the article and approved the submitted version.

## Funding

This study was supported by the National Natural Science Foundation of China (No. 81772923), the Science Research Project of Hunan Province (No. B20180661), the Scientific and Technology Department of Hunan Province (No. 2021SK4029), the Key Laboratory for Molecular Diagnosis and Precision Medicine in Hengyang (No. 202010041574). The funding agencies had no role in study design, data collection and analysis, preparation of the manuscript, or decision to publish.

## Conflict of Interest

The authors declare that the research was conducted in the absence of any commercial or financial relationships that could be construed as a potential conflict of interest.

## Publisher’s Note

All claims expressed in this article are solely those of the authors and do not necessarily represent those of their affiliated organizations, or those of the publisher, the editors and the reviewers. Any product that may be evaluated in this article, or claim that may be made by its manufacturer, is not guaranteed or endorsed by the publisher.
